# Supplementary material for: Complexome profiling of the Chlamydomonas psb28 mutant reveals TEF5 as an early PSII assembly factor
Source: Plant Cell. 2025 Mar 17;37(6):koaf055. doi: 10.1093/plcell/koaf055 (PMC12164590; doi:10.1093/plcell/koaf055)
Supplement: koaf055_Supplementary_Data [file koaf055_supplementary_data.zip › psb28_interactive.zip!/plots/intensity/qualityPlots/peptides/NUO5_Cre10.g450400.t1.2.html]

### Cre10.g450400.t1.2

Trivial name: NUO5  
SeqCoverage [median]: 45.40  
Mass: 30.61 kDa  
  
 

| Sequence | MissedCleavage | Mass [Da] | wtIntensity | muIntensity | Score |
| --- | --- | --- | --- | --- | --- |
| DGETTLTGAPR | 0 | 1116.54 | 14595.49 | 11387.76 | 209.21 |
| DIVNILDTIK | 0 | 1142.65 | 10438.89 | 0.00 | 133.99 |
| DIVNILDTIKK | 1 | 1270.75 | 7847.65 | 0.00 | 102.06 |
| DTPENNAATSFEFSEATLK | 0 | 2070.94 | 0.00 | 3405.86 | 80.59 |
| GGKPKPGSQYR | 2 | 1173.63 | 615.69 | 4186.78 | 90.65 |
| GVSGFEYIYYEDLTPK | 0 | 1879.89 | 3778.04 | 0.00 | 144.10 |
| IEEAITK | 0 | 802.44 | 29626.46 | 8813.19 | 165.11 |
| LKAEPAGAVHGGEK | 1 | 1362.73 | 17643.29 | 15460.49 | 162.69 |
| LLDMAPIR | 0 | 927.52 | 26434.80 | 16432.06 | 114.78 |
| LQGSQKIEEAITK | 1 | 1443.79 | 0.00 | 3281.79 | 42.21 |
| PGSQYR | 0 | 706.34 | 0.00 | 1308.68 | 95.79 |
| VVNDIIAR | 0 | 898.52 | 42691.97 | 34666.77 | 141.52 |
| VYEVATFYTMFNR | 0 | 1639.77 | 5287.46 | 0.00 | 140.49 |
| WVPKDGETTLTGAPR | 1 | 1626.84 | 3836.02 | 5787.31 | 66.28 |
|  |  |  | 162795.8 | 104730.7 |  |
